# Different land-use types equally impoverish but differentially preserve grassland species and functional traits of spider assemblages

**DOI:** 10.1038/s41598-021-89658-7

**Published:** 2021-05-13

**Authors:** Carolina M. Pinto, Pamela E. Pairo, M. Isabel Bellocq, Julieta Filloy

**Affiliations:** Departamento de Ecología, Genética Y Evolución, FCEN, Universidad de Buenos Aires - IEGEBA, CONICET, Ciudad Universitaria, Pab 2, piso 4, C1428EHA Buenos Aires, Argentina

**Keywords:** Ecology, Biodiversity, Community ecology

## Abstract

Land-use change is one of the major drivers of biodiversity loss by introducing environmental modifications, which excludes native species unable to adapt to the novel conditions. Grasslands are among the most threatened biomes; understanding the influence of different land-use types on native species is crucial to achieving sustainable management policies. We hypothesized that land-use types that partially conserve the original vegetation cover would show higher taxonomic and functional diversity and similarity with native assemblages than land-use types that replace the original vegetation cover. We compared the taxonomic and functional alpha and beta diversity of spider assemblages between soybean crops, eucalypt plantations, and cattle fields with seminatural grasslands. Through null models, we assessed the standardized effect sizes to test differences in the strength of environmental filtering among land-use types. Environmental changes introduced by different land-use types resulted in assemblages differentiated in species and trait composition, taxonomically and functionally impoverished with respect to seminatural grasslands. All land-use types drove species replacement and trait loss and replacement of grassland spiders. Each land-use showed a characteristic species and trait composition. Most of the grassland traits were not lost but were under or over-represented according to the land-use type. Only in soybean crops the formation of spider communities would be mainly driven by environmental filtering. Changes in land-use decreased species diversity and modified the composition of spider species and functional traits leading to differentiated spider assemblages. As spider species and traits varied among land-uses, a mitigation measure against grasslands biodiversity loss could be the development of productive landscapes with a mosaic of land-use types, as each of them would provide microhabitats for species with different requirements. Because land-use types mainly led to the rearrangement of grassland functional trait values, most of spider functions might be conserved in mosaics of land-use types.

## Introduction

Land-use change is one of the primary drivers of biodiversity loss and alteration of ecosystem functioning across the terrestrial surface, especially in grasslands^1^. Throughout human history, grasslands have been intensely modified and fragmented by agriculture, afforestation, urbanization, or grazing^2^. Grasslands cover around 40% of earth’s surface^3^, and are crucial as repositories of biodiversity^2^. However, the protected areas destined to grasslands reserves are insufficient for conservation purposes. Thus, deepening our knowledge regarding biodiversity responses to different land-use types developing in grasslands is central for achieving territorial planning and management strategies aimed to preserve native biodiversity and ecosystem functioning.

Different human activities typically lead to a variety of habitat types due to differential modifications of original microhabitats. Those changes in habitat conditions typically lead to non-random species loss^4,5^ resulting in poorer, functionally redundant, communities as functionally unique species tend to be lost^6^. Thus, biological assemblages in disturbed habitats are expected to show nested composition of species holding some functional traits of those occurring in less disturbed habitats^7^. Nonetheless, differences between habitats may include new combinations of environmental variables (i.e., new microhabitats) allowing new specific trait–environment relationships which may lead to changes. In such a case, a complete replacement of species and traits should be expected if a completely different habitat is set. Both types of changes in taxonomic or functional assemblage composition can be assessed by accounting for nestedness and turnover components of beta diversity estimation^8^. Thus, we expect the primacy of nestedness over turnover for those land-use types leading to microhabitat loss with respect to the original habitat. Conversely, we expect the primacy of turnover over nestedness for those land-use types that provide a new range of microhabitats.

Three processes have been proposed to shape species assemblages: dispersal, environmental filtering and biotic interactions^9^. When the abiotic environment is the main selective force, environmental filtering would exclude species unable to tolerate the conditions at a given location, while those species able to survive would share common traits associated with their abiotic requirements. This logic leads to some of the most widely tested predictions in environmental filtering studies; species at a site would exhibit phenotypic convergence, relative to a null expectation based on random sampling from the species pool^10^. When species interactions are the main determinant factor (e.g., competition) these interactions are supposed to increase within‐community functional diversity by limiting the similarity among coexisting species traits, giving rise to functional trait divergence^11,12^. Thus, as environmental filtering has been proposed as the main driving force of the distribution of trait values in human-modified ecosystems^13^, we expect different degrees in spider trait convergence or even divergence in land-use types depending on the environmental dissimilarity between the anthropic and the native habitats.

It has been shown that functional diversity determines ecosystem functioning rather than species numbers *per se*^14^. Predators play a vital role in ecosystems by maintaining the structure and stability of ecological communities^15^. Spiders, as generalist predators, are essential in trophic networks because of their high abundance, biomass and species diversity^16^. Previous studies concerning epigeal spiders have shown that as agricultural management intensity increases, spider diversity decreases^17^. However, mismatches between taxonomic and functional diversity patterns may occur as a consequence of species traits selection by the different characteristics of the modified habitats (e.g. resources availability and abiotic conditions)^18^. Thus, land use types setting open habitats are expected to promote greater and ectotherm species in warm conditions^19^. Also, a wind-dispersal ability such as ballooning would be favored^20^. Regarding hunting mode, a simplification in the stratification of the vegetation may be detrimental for web-building spiders^21^. Light permeability through vegetation is a key determinant of spiders circadian activity; close habitats would favor nocturnal dark-adapted species rather than open habitats^22^. Thus, assessing individually the changes in particular functional traits can shed light on to the role of the different land-use types in preserving native ecosystem functions.

Our goal was to disentangle the processes driving diversity of spider assemblages in different land-use scenarios developing in a grassland biome. We based our study on a general hypothesis stating that those land-use types that partially conserve the original vegetation cover show higher taxonomic and functional diversity and similarity with native assemblages than those land-use types that replace the natural vegetation cover; the latter will experience the replacement of native species by alien species, decreasing the similarity with the native assemblages. The different environmental filters imposed by each land-use type lead to assemblages differentiated in their trait composition. Thus, we predict (1) nestedness to underlie taxonomic and functional dissimilarity between cattle fields and seminatural grassland assemblages; (2) turnover to underlie taxonomic and functional dissimilarity between monocultures (i.e. soybean crops and tree plantations) and seminatural grassland assemblages; (3) trait convergence to be more prevalent in cattle fields compared to soybean crops, and tree plantations assemblages; (4) a directional change in individual functional traits when comparing each land use type with the seminatural grasslands. Regarding the main individual traits considered to estimate functional diversity of spiders communities (Table 1) we expect 4.1) an increase in spiders body size in soybean crops, and a decrease in tree plantations^23^; 4.2) an increase in ballooning tendency in soybean crops^24^; 4.3) a decrease in web-building spiders in cattle fields and tree plantations^25^; and 4.4) an increase in nocturnal spiders in tree plantation^22^.

**Table 1:** Spiders functional traits specification with the respective environmental response association.

Trait	States	References	Environmental response
Mean body size	Cephalothorax length mm (continuous)	Taken from the individuals caught	Associated with metabolic rate^66^; larger spiders inhabit warm and dry environments and smaller in cool and moist ones^67^
Hunting mode	Web–Ambush–Active hunting	Cardoso et al. (2011)	Associated with the habitat structure^25^. Soil-dwelling spiders increase with the litter layer and web-building spiders are linked to the configuration of the vegetation^21^
Dispersal ability	Ballooning common (yes–no)	Bell et al. (2005)	Associated with species colonization ability; typical performed by spiders from open, unstable habitats^24^
Circadian activity	Diurnal or Nocturnal	Cardoso et al. (2011)	Canopy cover can influence the circadian rhythm of arthropods^22^. More nocturnal activity in closed habitats vs in open ones

## Materials and methods

### Study area and sampling design

**Figure 1:**
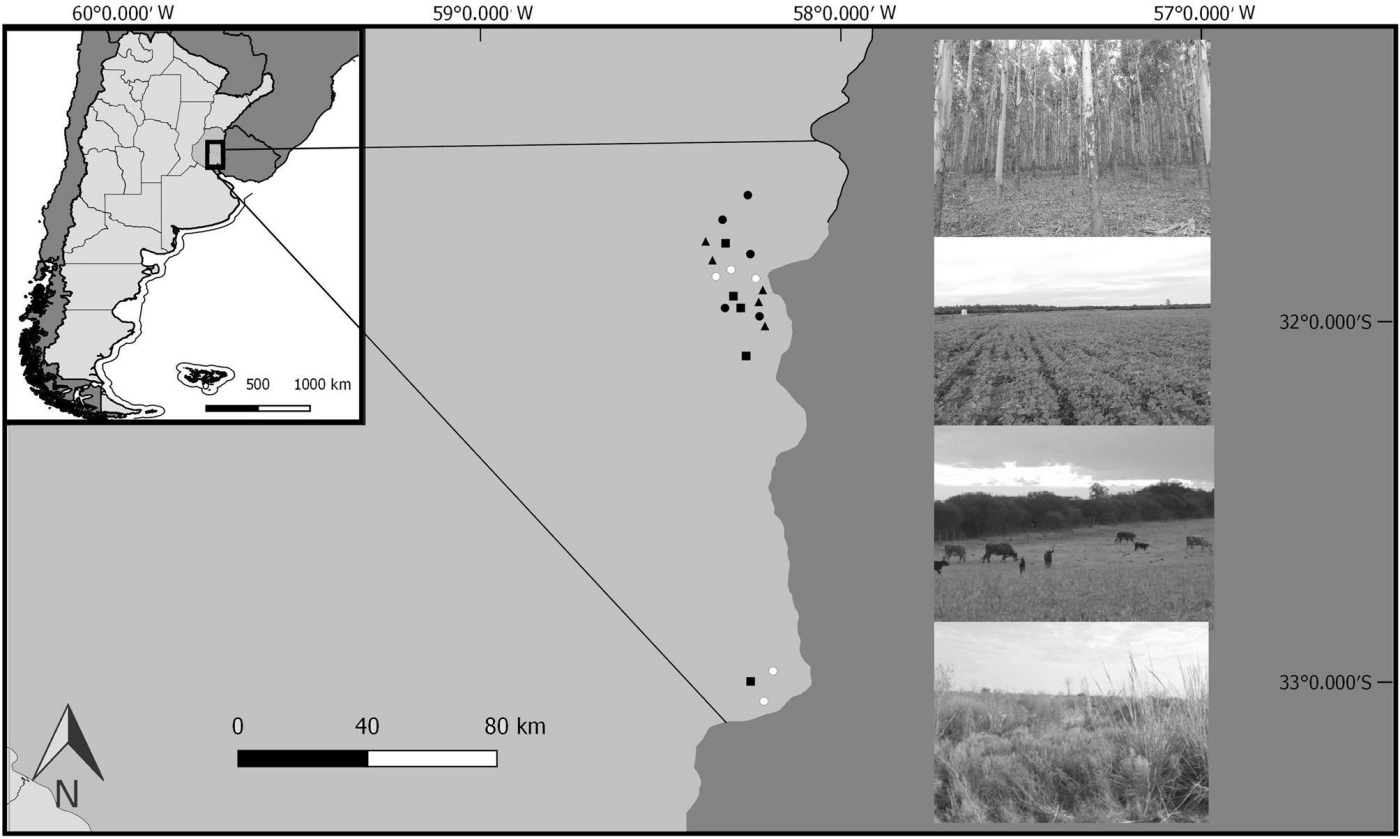
Study sites in the central-eastern Argentina. Black circles represent eucalypt plantations, white circles are protected natural grasslands, triangles are cattle fields and squares are soybean crops fields. On the right there is a representative picture of each land-use type, from the top to the bottom first eucalypt tree plantation, then soybean crop field, cattle field and natural grassland.

The study was conducted in central-eastern Argentina (31–33°S and 58–59°W, Fig. 1). The climate is humid temperate with precipitations occurring all year round. The dominant vegetation is a grass steppe dominated by *Stipa*, *Piptochaetium*, *Poa*, *Eragrostis*, and *Aristida*, wild straws (*Melica*), wisps (*Briza*), and bromes (*Bromus*)^26^. Traditionally, this region was devoted to livestock production. Since the 1990s livestock areas have been replaced by agriculture^27^. The study area is dominated by large extensions of monocultures crops, usually wheat in winter and soybean in summer. Between successive summer soybean crops, the land may remain at rest or livestock may feed on the non-implanted pastures. Eucalypt plantations have also replaced livestock fields^28^. *Eucalyptus grandis* is the dominant plantation tree in the region, but also pine (*Pinus* sp.) is planted^29^. Plantations are large-scale monocultures covering hundreds of hectares with stands which have little understory vegetation^5^ and large amounts of leaf litter.

We sampled spiders in soybean crops, eucalypt tree plantations, cattle fields, and in protected areas (seminatural grasslands), the latter used as a reference of the native habitat. The sample design consisted of 20 study sites (about 350–800 m^2^), five sites (i.e., replicates) per habitat type. The study sites were separated by a mean distance of 45 km, from 500 m to 150 km. We selected mature stands (more than 9 year old) of *Eucalyptus grandis* to avoid heterogeneity among replicates^30^. Spider sample stations were set up at least at 50 m inside each study site to avoid edge effects^31^.

### Environmental variables

To environmentally characterize the different land-use types, we estimated the percentage of land cover by shrubs, herbs, leaf litter and bare soil. We also measured the mean height, maximum height and density of the herbaceous stratum and the leaf litter depth^32^. At each sampling site, we visually estimated the coverage percentages following Kent and Coker (1992) in three quadrants of 4 m × 4 m. At each quadrant, we measured the maximum vegetation height, the mean vegetation height of the herbaceous stratum from 10 points taken randomly, and the litter depth in the middle of each quadrant. We calculated vegetation density in the lower stratum using a digital camera positioned approximately 1 m from a white cloth of 1 × 1 m used as a background screen and we took a picture capturing the vegetation in the meedle^33^. We estimated the percentage covered by vegetation by transforming the photographs into black for vegetation and white for background screen, and quantifying them with the ImageJ program^34^.

### Spider sampling and identification

Spiders were sampled using pitfall traps to collect ground mobile spiders and an entomological vacuum (G-vac) method to catch less mobile ones from the herbaceous strata^35^. At each study site (sample unit) three pitfall traps (diameter = 9 cm, depth = 10 cm) were placed 10 m apart from each other^36^. Traps were half filled with propylene glycol and water (30:70). A plastic roof was placed 10 cm above the trap to prevent flooding and evaporation^37^. Pitfall traps worked for 6 weeks, 3 in spring (November 2015) and 3 in summer (January 2016). The G-vac method was used to suck spiders from the herbaceous strata in a 1 × 1 m square for 1 min using a vacuum with a 110 cm long and 12 cm wide tube. On each site and sampling period we collected five subsamples diurnally and separated at least by 10 m^38^. The material per site was pooled together and conserved in ethanol 70%. Adult spiders were identified to species or morphospecies using taxonomic keys and assistance of arachnologists from the “Museo Argentino de Ciencias Naturales Bernardino Rivadavia”, Buenos Aires, Argentina. Spiders represented by only one individual (singletons) were excluded from all analyses to avoid including accidental captures^39^.

### Spider traits

Spiders were characterized according to four widely accepted traits that typically reflect spiders adaptation to environmental conditions: body size, hunting mode, dispersal ability and circadian activity^36,40^ (Table 1). For the estimation of body size (i.e., cephalothorax length) of each species two to four specimens were used; measurements were done with a 0.01 mm ocular micrometer mounted on a Carl-Zeiss Discovery V8 stereo-microscope. Hunting mode, dispersal ability and circadian activity states for each species were obtained from literature (Table 1).

### Data analysis

To describe the environmental differences among sites, we performed a principal component analysis (PCA)^41^ using R package vegan^42,43^. Since environmental variables were not dimensionally homogenous, they were standardized, and a correlation matrix was used for the analysis.

#### Taxonomic and functional diversity comparison among land-use types

To analyze spider taxonomic and functional diversity change due to different land-use types, data from pitfall traps and G-vac within each study site were pooled together. For statistical analyses, species richness was expressed as the number of species per sampling site, and functional diversity as the FDis index estimated per sample site. We calculated the alpha functional diversity as the FDis multi-trait index^44^. Life-history trait data for dispersal, circadian activity and hunting mode were coded categorically and body size as a quantitative variable for the index calculation. We also used the community-weighted trait means (CWM onwards) to measure changes in the functional composition of spider assemblages driven by land-use types^45^. To calculate the CWM values for each category, categorical traits were treated as independent binary variables^46^.

We ran Generalized Linear Models (GLM) with land-use type as the predictor variable (four levels: the three land-use types and the seminatural grasslands) and the species richness and the FDis index as response variables using R package stats. The Poisson distribution with the logarithm as link function was used for species richness (after meeting for no overdispersion) and the Gaussian distribution with the identity as link function was used for the FDis (after meeting for linearity and homoscedasticity)^47^. Then, Tukey post-hoc comparisons were run to test for differences between land-use types and the seminatural grasslands (used as reference) using emmeans package in R.

#### Taxonomic and functional similarity among land-use types

To analyze changes in species and functional traits composition among land-use types, we calculated the dissimilarity of spider assemblages between each pair of land-use and seminatural grassland sites. To find out if the dissimilarities were due to replacement or loss of species or traits, we obtained nestedness and turnover components following Baselga (2010) and Villéger et al. (2013). For taxonomic dissimilarity Jaccard index was used. For functional trait dissimilarity, we first obtained the Gower distance between pairs of species based on the table of trait data. Then, we ran a principal coordinate analysis (PCoA) and we used the four resulting axes as continuum functional dimensions to delimit the functional space of each site. Finally, the volumes of multivariate trait space shared by two sites were obtained as the functional similarities between pairs of sites^48^. Taxonomic and functional pairwise beta diversity measures were calculated. Then, to test the differences in the nestedness and turnover components among land-use types we ran permutational multivariate analyses of variance (PERMANOVA)^49^ using the vegan package of R (adonis function) and performed post-hoc pairwise comparisons using the RVAideMemoire package of R (pairwise.perm.manova function) (perm = 9999) after checking meeting all assumptions.

#### Response of individual species and traits to land-use type

We identified the species involved in the taxonomic dissimilarity patterns by running non-metric multidimensional scaling (NMDS) with Jaccard dissimilarity measures using vegan package of R (metaMDS function). We utilized the scores from axis 1 and 2 of the NMDS ordination to select the three spider species that showed the strongest association with each land-use type. Then, to examine the spider trait composition changes among land-use types, we compared CWM values between land-use types and seminatural grasslands. We ran GLMs in R with land-use type as the predictor variable (four levels: the three land-use types and the seminatural grasslands) and the CWM of each functional trait as the response variable. The Gaussian distribution with the identity as link function was used for the CWM of the spider body size, and the binomial and quasibinomial distribution with the logarithm as link function were used for CWM of categorical variables^47^. Post-hoc comparisons were run to test for differences between land-use types and the seminatural grasslands.

#### Formation processes of spider communities in land-use types

To assess the environmental filtering process in all land-use types, we compared the observed FDis and the FDis expected by chance for each study site. We ran null models by generating 999 random assemblages per site, using the complete list of species collected^50^. Each random assemblage maintained the same number of species observed per site. To analyze the difference between the observed FDis and that expected by chance for each land-use type, the standardized effect sizes of FDis (SESFDis) was calculated^50^. SES negative values indicated functional convergence (i.e., loss of functional diversity leading to functional redundancy of spiders assemblages) while positive values indicated functional divergence (i.e., gain of functional diversity leading to an increase in spider species functional differentiation)^51^. To estimate whether the SESFDis means were negative or positive, t-tests were performed using stats package of R (t.test function).

## Results

We recorded a total of 2085 individuals (823 adults) from 105 spider species or morphospecies (24 families) (Appendix, Table A1) in the 20 sampled sites. Sixty-six species were caught in seminatural habitats while 36 were found in soybean, 40 in cattle fields and 34 in eucalypt plantations. Approximately 29% of species were exclusively from seminatural grasslands. The most abundant families were Lycosidae (20.5%), Theridiidae (17.8%) and Linyphiidae (15.7%) considering only adults. The most frequently observed spider species was *Pardosa flammula* (Lycosidae, 8% of adults), followed by *Erigone* sp1 (Linyphiidae, 7% of adults).

**Figure 2:**
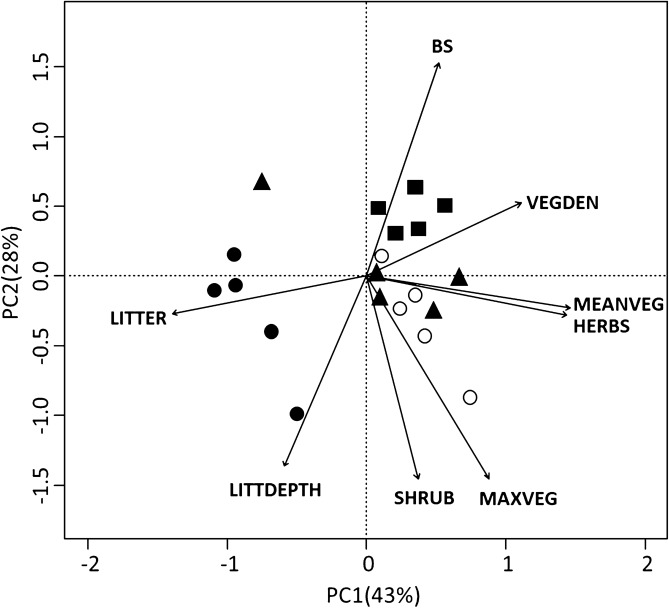
Biplot representing the first (PC1) and second (PC2) axes of principal component analysis, showing ordination of sites according to environmental variables measured in cattle fields (triangles), soybean crops (squares), seminatural grasslands (white circles), and tree plantations (black circles). Environmental variables are percentage coverage by shrubs (SHRUBS), herbaceous vegetation (HERBS), leaf litter (LITTER), and bare soil (BS), the mean vegetation height (MEANVEG), the maximum vegetation height (MAXVEG), vegetation density (VEGDEN), and the leaf litter depth (LITTDEPTH).

The PCA based on environmental variables showed that two components explained 71% of the environmental variation (Fig. 2). The first component (43%) differentiated tree plantations and one cattle field from the rest of the sites. The second component (28%) mainly differentiated soybean crops from the other sites. Seminatural grasslands and cattle fields were characterized by high coverage of herbs and shrubs, and high mean and maximum vegetation height. Tree plantations were characterized by high cover and depth of leaf litter, while soybean crops showed high percentage of bare soil and high vegetation density.

### Taxonomic and functional diversity comparison among land-use types

**Figure 3:**
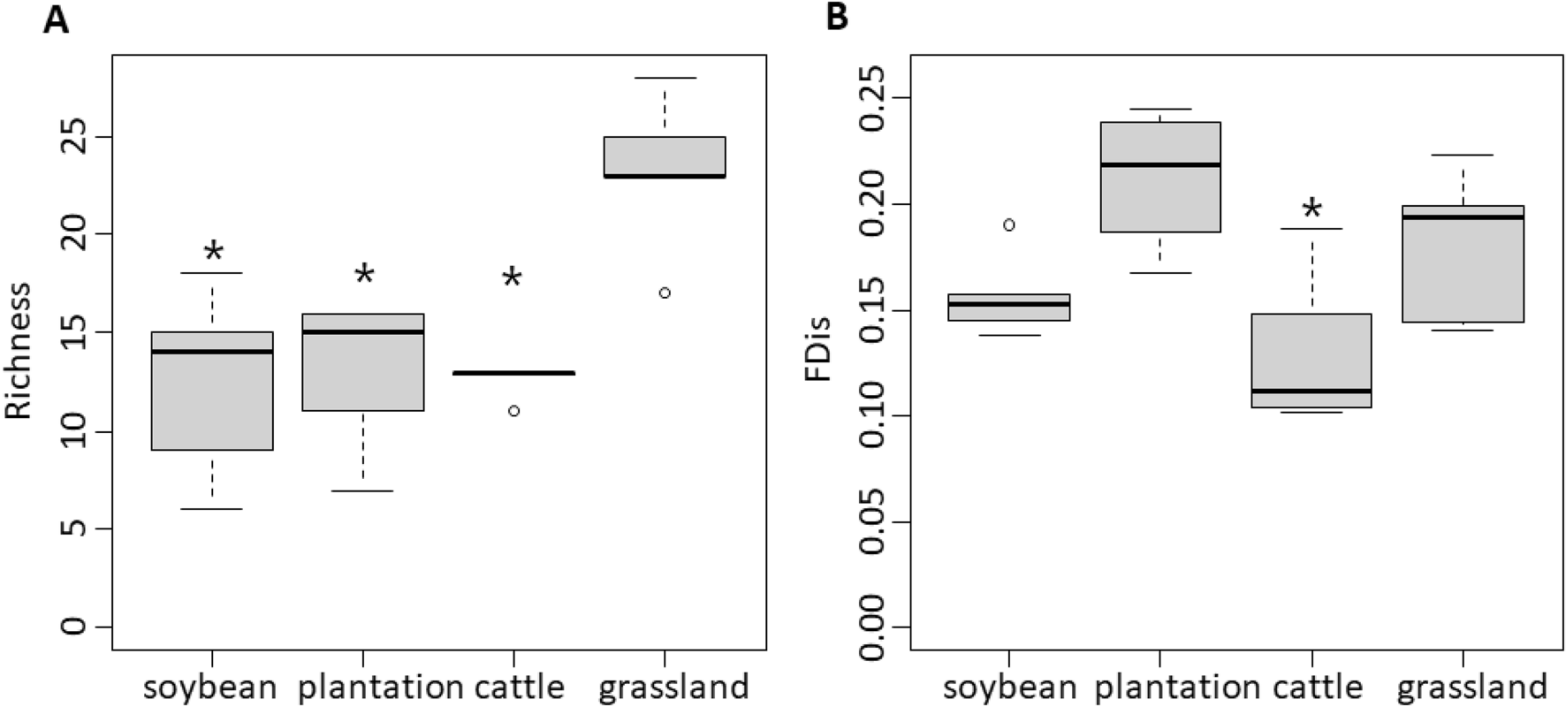
Boxplot of spider richness (**A**) and functional diversity (**B**) in different land-use types and seminatural grasslands. The central line represents the median, boxes include the third and first quartiles, whiskers show maxima and minima, and circles represent suspected outliers. Asterisks represent significant differences between the means of land-use types and seminatural grasslands (*p* < 0.05).

Spider richness differed among land-use types and seminatural grasslands (*X*^2^_*3,16*_ =  − 34.88, *p* < 0.001), with greater species richness in seminatural grasslands than in cattle fields (*Z* =  − 3.90, *p* < 0.001), tree plantation (*Z* =  − 3.71, *p* < 0.001), and soybean crops (*Z* =  − 3.98, *p* < 0.001) (Fig. 3A). Functional diversity also differed among land-use types and seminatural grasslands (*X*^2^_3,16_ = 16.75, *p* = 0.001), with lower FDis in cattle fields than in seminatural grasslands (*Z* = 2.40, *p* = 0.04) and no statistical differences between soybean crops and tree plantation with the seminatural grasslands (soybean vs grasslands: *Z* =  − 1.15, *p* = 0.51; plantations vs grasslands: *Z* = 1.52, *p* = 0.30) (Fig. 3B).

### Taxonomic and functional similarity among land-use types

**Figure 4:**
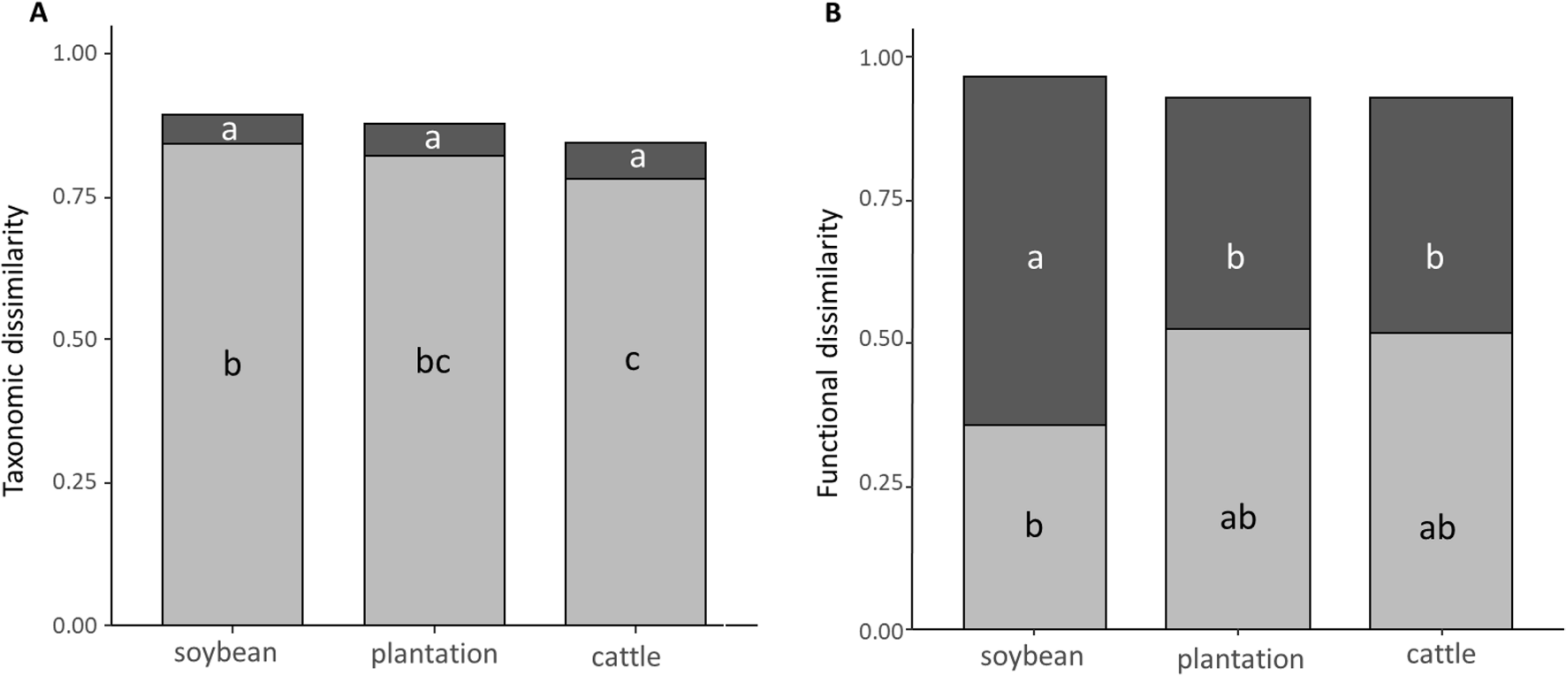
Mean of the total, the nestedness (dark grey), and turnover components (light grey) of taxonomic (A) and functional (B) dissimilarities between land-use types and seminatural grasslands. The different letters represent statistical differences among the components (*p* < 0.05).

The species composition of the three land-use types was highly dissimilar compared to seminatural grasslands, being the total dissimilarity CI_95_ = 0.87 ± 0.09 for soybean crops, CI_95_ = 0.84 ± 0.06 for tree plantations, and CI_95_ = 0.0.81 ± 0.08 for cattle fields. The PERMANOVA analysis showed that the taxonomic turnover and nestedness components differed among land-use types (*F* = 3.19, *p* = 0.04), being turnover higher than nestedness for the three land-use types (Appendix, Table A2) (Fig. 4A). In addition, turnover was higher in soybean crops than in cattle fields, but no differences were found in the turnover component between soybean crops and tree plantations, and between tree plantations and cattle fields. Differences among nestedness component were not significant (Appendix, Table A2) (Fig. 4A).

The total functional dissimilarity between each land-use type and seminatural grasslands was high, CI_95_ = 0.96 ± 0.03 for soybean crops, CI_95_ = 0.93 ± 0.04 for tree plantations, and CI_95_ = 0.93 ± 0.03 for cattle fields, with significant differences between the functional turnover and nestedness components among them (*F* = 4.90, *p* = 0.01) (Fig. 4B). Turnover was similar among land-use types. For soybean crops, the turnover component was lower than the nestedness component (Appendix, Table A2). Nestedness was higher in soybean crops than in tree plantations and, than in cattle fields (Appendix, Table A2), and no statistical differences were found for the nestedness component between tree plantations and cattle fields (Fig. 4B).

### Response of individual species and traits to land-use type

**Figure 5:**
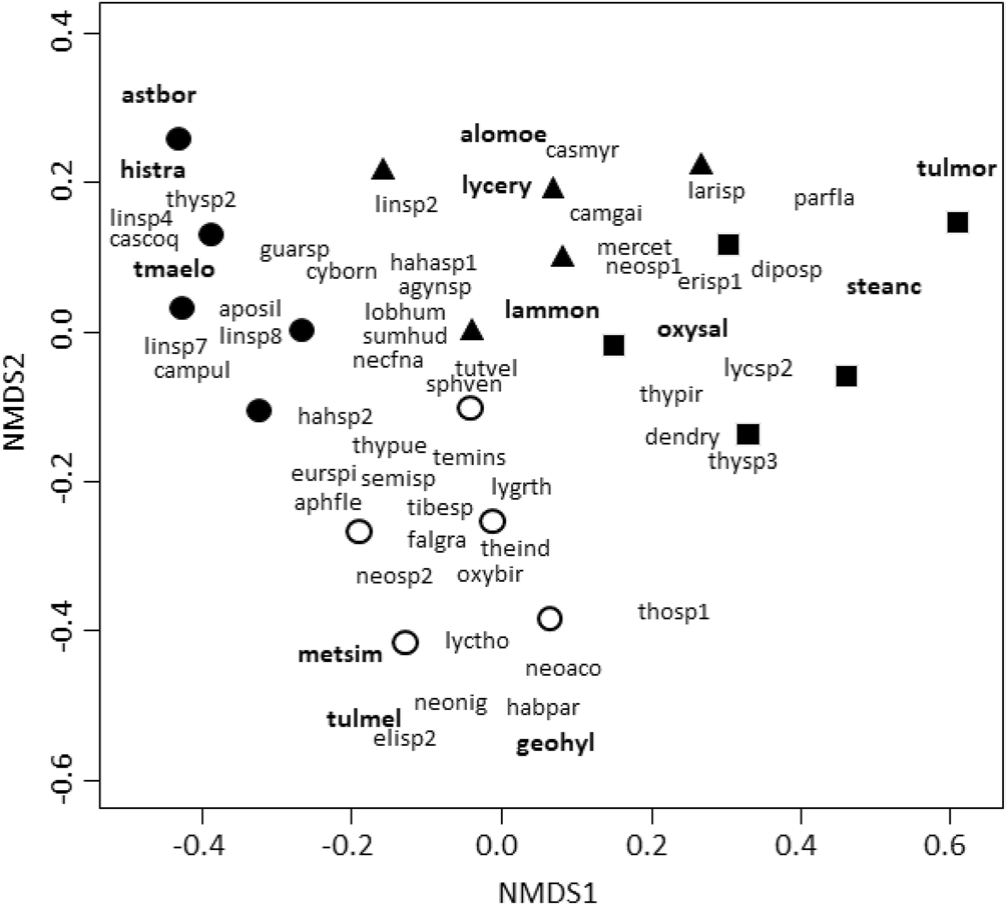
Nonmetric multidimensional scaling (NMDS) ordination in two dimensions of land-use sites and seminatural grasslands based on the similarity of species composition. Species are represented by their name’s abbreviations (Appendix, Table A1), black circles are tree plantation sites, white circles are seminatural grassland sites, triangles are cattle fields and squares are soybean crops. In bold species that presented the strongest association with the different land-use types based on NMDS scores.

The NMDS analysis ordered sites into four groups (Fig. 5). Based on species scores (Appendix, Table A3), each land-use type was characterized by different spider species: *Hisukattus transversalis* (histra), *Tmarus elongatus* (tmaelo) and *Asthenoctenus borellii* (astbor) in tree plantations; *Alopecosa moesta* (alomoe), *Lycosa erythrognatha* (lycery) and *Laminacauda montevidensis* (lammon) in cattle fields; *Tullgrenella morenensis* (tulmor), *Steatoda ancorata* (steanc) and *Oxyopes salticus* (oxysal) in soybean crops; and *Geolycosa hyltonscottae* (geohyl), *Metaltella simoni* (metsim) and *Tullgrenella melanica* (tulmel) in seminatural grasslands.

**Figure 6:**
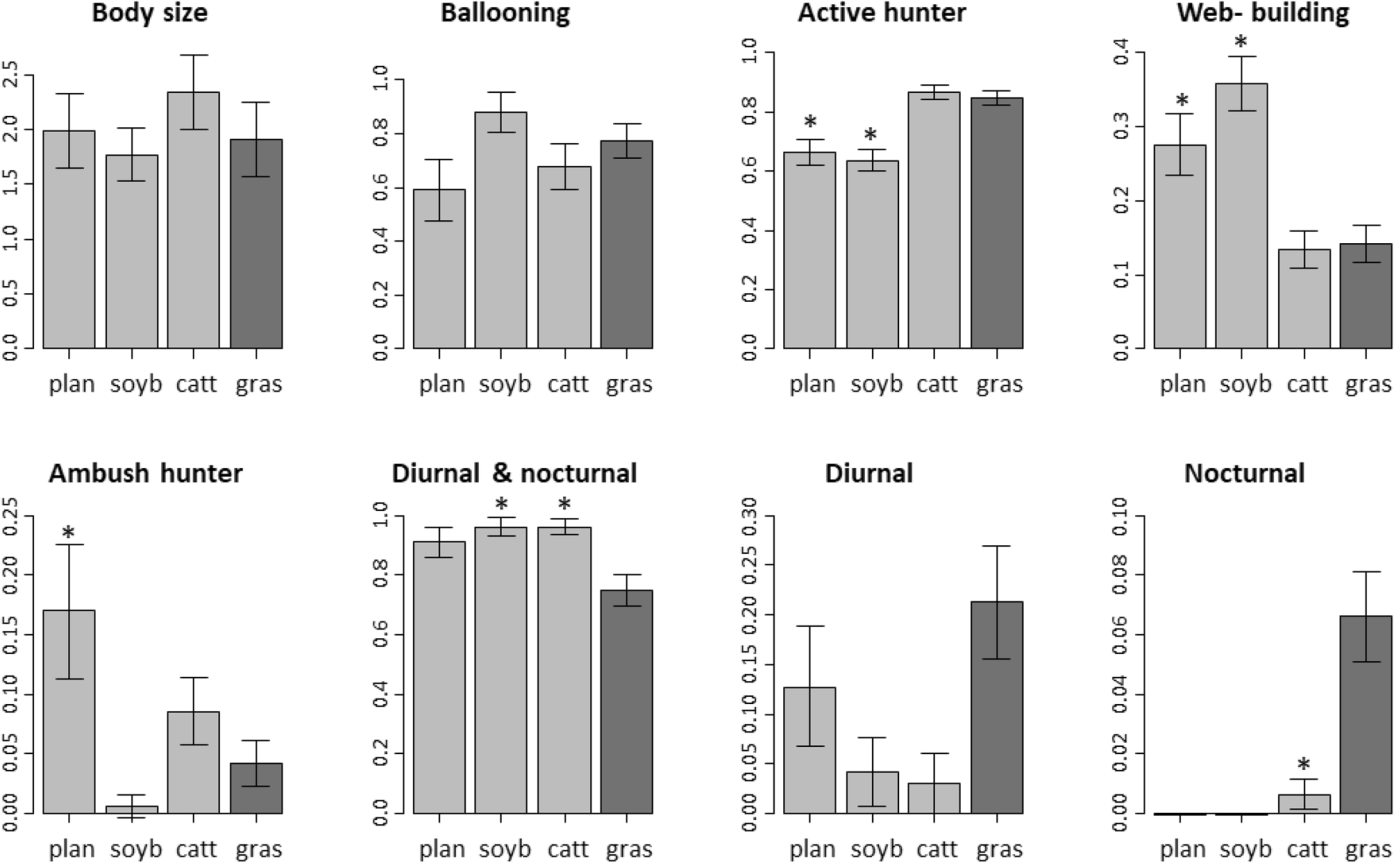
Community weighted means (mean ± standard error) of the functional traits of spider assemblages located in tree plantations (plan), soybean crops (soyb), cattle fields (catt), and seminatural grasslands (gras). In each graphic, asterisks represent significant differences between the means of land-use types and seminatural grasslands (*p* < 0.05).

The CWM of spider traits varied among land-use types compared to seminatural grasslands for hunting strategy and circadian rhythm, but not for body size and the ability to disperse by ballooning (Fig. 6). Regarding hunting strategy, active hunters were less represented in soybean crops and tree plantation than in seminatural grasslands (*X*^2^_3,16_ = 36.97, *p* < 0.001; soybean vs grasslands : *Z* =  − 4.08, *p* < 0.001; plantations vs grasslands: *Z* =  − 4.19, *p* < 0.001); web-building spiders were more represented in soybean crops and tree plantations than in seminatural grasslands (*X*^2^_3,16_ = 32.47, *p* < 0.001; soybean vs grasslands : *Z* = 4.21, *p* < 0.001; plantations vs grasslands: *Z* = 3.71, *p* < 0.001); ambush hunters were more represented in tree plantations than in seminatural grasslands (*X*^2^_3,16_ = 16.71, *p* < 0.001; *Z* = 2.88, *p* = 0.01). As regards the circadian rhythm of spider assemblages, cattle fields and soybean crops presented higher proportions of spiders that are diurnal and nocturnal than seminatural grasslands (*X*^2^_3,16_ = 16.47, *p* < 0.001; cattle vs grasslands : *Z* = 2.64, *p* = 0.02; soybean vs grasslands: *Z* = 2.46, *p* = 0.04); cattle fields presented lower proportions of exclusively nocturnal spiders than seminatural grasslands (*X*^2^_3,16_ = 30.47, *p* < 0.001; *Z* =  − 2.47, *p* = 0.04), and no exclusively nocturnal spiders were found in tree plantation and soybean crops (Fig. 6); the proportions of exclusively diurnal species did not differ among land-use types (*X*^2^_3,16_ = 7.39, *p* = 0.06).

### Formation processes of spider communities in land-use types

**Figure 7:**
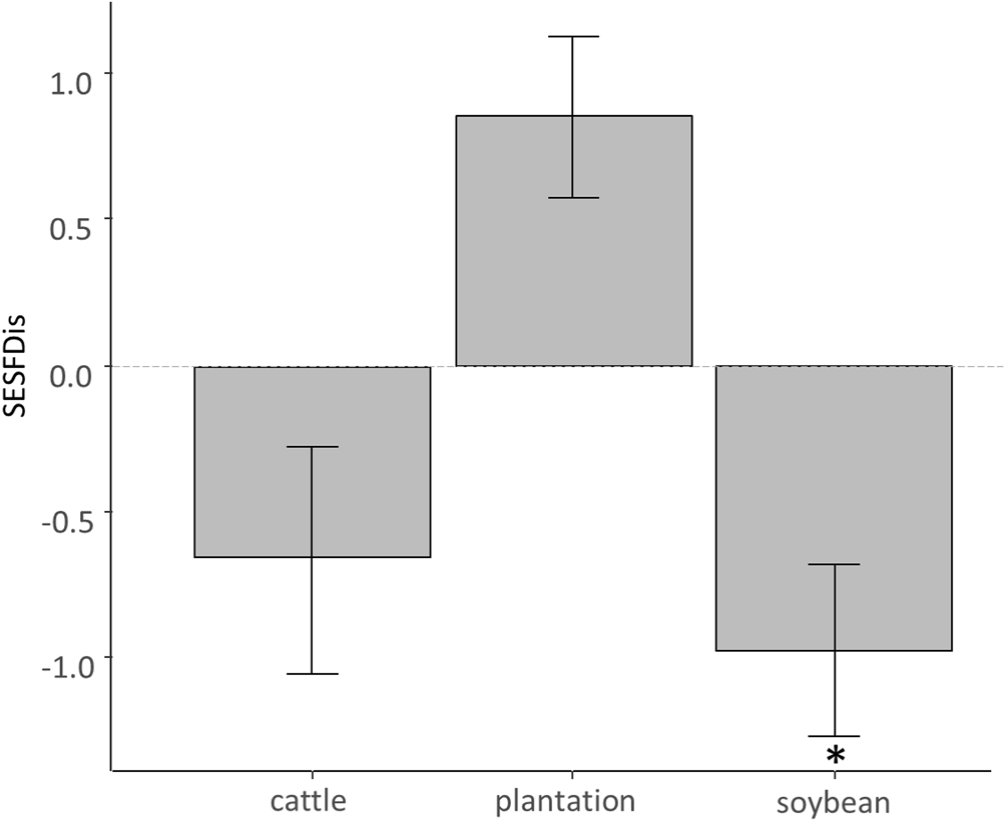
Standardized effect size of FDis index (SESFDis) (mean ± standard error) for different land-use types. SESFDis = 0 indicates the expected value when assemblages follow the null model. Asterisks represent significant departures from 0 obtained by t-tests (*p* < 0.05).

Tests performed to assess whether SESFDis were negative or positive indicated that for cattle fields and tree plantation the SESFDis values did not differ from the null expectations (*t*_*4*_ =  − 0.50, *p* = 0.64 and *t*_*4*_ = 2.29, *p* = 0.08, for cattle fields and tree plantations respectively). For soybean crops the SESFDis was significantly lower than zero (*t*_*4*_ =  − 4.57, *p* = 0.01) (Fig. 7).

## Discussion

Overall, our results showed that land-use types introduced different environmental modifications, however, all land-uses led to assemblages impoverished in grassland spider species and traits irrespective of their environmental similarity with the native habitat. Likely, at the microhabitat scale perceived by spiders, the magnitude of environmental change due to the different land-use types was equivalent. Besides, our results revealed that changes introduced by each land-use type were qualitatively different. Soybean crops retained more grassland spider traits than other land-use types and favored web-building spiders. Tree plantations enhanced ambush hunters while only cattle fields harbored nocturnal species. The three land-use types led to grassland species replacement and loss and replacement of functional traits. However, only in soybean crops, the environmental filtering seemed to be the main process involved in assembly formation. Possibly, the neutral patterns found in tree plantations and cattle fields were due to the compensation of opposite trends driven by environmental filtering and niche differentiation processes acting simultaneously (i.e. functional convergence and divergence, respectively).

### Taxonomic and functional diversity comparison among land-use types

We found that soybean crops, eucalypt plantations, and cattle fields equally reduced species richness but not the functional diversity of grassland spider assemblages. As observed in previous studies, environmental changes introduced by human land-uses strongly limit the number of species able to tolerate the new habitat conditions^52,53^ and only those species holding functional traits reflecting adaptations to the modified habitats will be able to persist^9^. Except for cattle fields, land-use types did not decrease the functional diversity of spider assemblages compared to seminatural grasslands. The lack of functional diversity response in tree plantation and soybean crops showed that many of the lost species would be functionally redundant and might also be evenly distributed across the traits considered here. However, species loss and replacement could also lead to assemblages with less richness but no decrease in functional diversity due to rearrangements on functional trait values^54^.

### Taxonomic and functional similarity among land-use types; response of individual species and traits

Land-use types drove species composition shifts. Contrary to our expectations, the spider species composition of the three land-use types was highly dissimilar compared to protected grasslands and was mainly due to species replacement. As environmental conditions differed among land-use types, they might favor different spider species according with their habitat requirements. For example, in tree plantations, *Hisukattus transversalis* and *Asthenoctenus borellii* were more abundant, and have been previously found in implanted forests and other habitats closely linked to leaf litter and humidity conditions^55,56^. Also, *Alopecosa moesta* and *Laminacauda montevidensis*, highly abundant in cattle fields, have been found in protected areas and urban green areas^57,58^. *Tullgrenella morenensis* and *Steatoda ancorata*, which were more abundant in soybean crops, were also found in urban green areas and rice crops^58,59^. These species would be highly tolerant to anthropogenic disturbances and only depend on the herbaceous vegetation layer. Although vegetation cover in cattle fields was more similar to grasslands than other land-use types, at the microhabitat scale perceived by spiders, the magnitude of the environmental change introduced by all land-use types would be equivalent.

The land-use types induced changes in functional traits composition due to trait loss and replacement with respect to spider assemblages of seminatural grasslands. Overall, soybean crops were functionally more similar to grasslands than cattle fields and tree plantation. Possibly, the soybean vegetation layer provide a suitable structure for spiders adapted to grassland habitats^21^. While nocturnal spiders were only found in cattle fields and grasslands, most of the changes were due to the under or over-representation of grassland traits in the different land-use types. For example, active hunters decreased in tree plantations and soybean crops compared to grasslands, showing that this guild would be sensitive to changes in the vegetation cover as they depend on habitat architecture for concealment and prey location^60^. Web-building spiders increased in tree plantations and soybean crops compared to grasslands. The litter layer and fallen branches present on the ground of tree plantation and the high vegetation density in soybean crops may offer a variety of physical structures for webs attachment^61,62^. These results suggest that although an equivalent magnitude of the environmental change between land-use types and grasslands was perceived by spiders, the quality of those changes differed. Thus, functional traits modification could be driven by microhabitat loss and replacement^63^, being detrimental for those traits that relied on the lost microhabitats while species holding traits adapted to remaining and novel microhabitats would be favoured. However, some traits would not be affected by land-use habitat change (i.e., body size and ballooning). Likely, body size and ballooning might respond to microhabitat variables such as temperature^19^ and wind speed^64^, which would not show high variation among land-use types and natural grasslands.

### Formation processes of spider communities in land-use types

Regarding the functional traits considered here, functional convergence, evidence of environmental filtering^50^, only occurred in soybean crops. However, assemblages may also be composed of random subsets of functional traits from the regional pool. Possibly, the results obtained for tree plantations and cattle fields implied different processes that may be acting simultaneously. If niche differentiation and environmental filtering acted simultaneously with a similar strength, because these two processes lead to opposite patterns (i.e., functional divergence and convergence), these would explain a neutral net response for these land-uses. Niche differentiation has been commonly associated with the prevalence of competitive interactions^12^. However, in forest plantations niche differentiation could also occur due to the incorporation of novel forest structures, such as deep leaf mulch or woody structures, that would allow functionally distinct species to occupy novel available microhabitats^65^. While environmental filtering was claimed as the main driver of assemblage formation in anthropogenic habitats^6^, land-use change leading to novel environmental conditions may lead to other assembly processes over environmental filtering.

Environmental changes introduced by each land-use resulted in spider assemblages differentially composed regarding species and functional traits. Besides, environmental filtering may not be the main assembly process for all land-use types. Land-use types impoverished grassland spider assemblages. However, as spider species and traits were highly dissimilar among land-use types and overall retained about 70% of the species registered in grassland sites, the development of mosaics of different land-use types would favour the conservation of grassland spider diversity at the landscape scale. Finally, our results strongly suggest that patches of protected grasslands should always be part of the productive landscapes, as they would act as a refugee for those native species unable to adapt to disturbed habitats (i.e. land-use types).

## Supplementary Information

Supplementary Information 1.

Supplementary Information 2.

Supplementary Information 3.
