# Supplementary material for: Different land-use types equally impoverish but differentially preserve grassland species and functional traits of spider assemblages
Source: Sci Rep. 2021 May 13;11:10316. doi: 10.1038/s41598-021-89658-7 (PMC8119495; doi:10.1038/s41598-021-89658-7)
Supplement: Supplementary file 1 — Supplementary Information 1. [file 41598_2021_89658_MOESM1_ESM.docx]

**Different land-use types equally impoverish but differentially preserve grassland species and functional traits of spider assemblages**

Carolina M. Pinto^a^*, Pamela E. Pairo^a^, M. Isabel Bellocq^a,b^, Julieta Filloy^a^

^a^Departamento de Ecología, Genética y Evolución, FCEN, Universidad de Buenos Aires – IEGEBA, CONICET, Ciudad Universitaria, Pab 2, piso 4, C1428EHA, Buenos Aires, Argentina.

^b^M. Isabel Bellocq passed away on 9 July 2019.

* Corresponding author. Tel.: +5401156421009.

E-mail address: carolinapinto@ege.fcen.uba.ar - carolinap90@gmail.com (C.M. Pinto)

| **Table A1:**  Spider species and morphospecies collected with pitfall traps and G-vac in land use types and natural grasslands in central-eastern Argentina. | | | | |
| --- | --- | --- | --- | --- |
| **Family** | **Species and morfoespecies** | **Species abbreviations** | | **Reference** |
| **Actinopodidae** | *Actinopus* sp | actino | (Ferretti et al., 2010) | |
| **Anyphaenidae** | *Arachosia* sp | aracsp |  | |
|  | *Otoniela quadrivittata* | otiqua | (Brescovit, 1996) | |
| **Araneidae** | *Larinia sp* | larisp |  | |
| **Cheiracanthiidae** | *Cheiracanthium inclusum* | cheinc | (Edwards, 1958) | |
| **Corinnidae** | *Castianeira coquito* | cascoq | (Rubio et al., 2015b) | |
|  | Castianeira myrmecotypus | casmyr |  | |
|  | *Falconina gracilis* | falgra | (Bonaldo, 2000) | |
| **Ctenidae** | *Asthenoctenus borellii* | astbor | (Simó and von Eickstedt, 1994) | |
|  | *Isonectus ordinario* | isoord | (Polotow and Brescovit, 2009) | |
| **Desidae** | *Metaltella simoni* | metsim | (Leech, 1971) | |
| **Gnaphosidae** | *Almafuerte peripampasica* | almper | (Grismado and Carrión, 2017) | |
|  | *Apopyllus silvestrii* | aposil | (Platnick and Shadab, 1984) | |
|  | *Camillina pulcher* | campul | (Platnick and Shadab, 1982) | |
|  | *Camillina galianoae* | camgal | (Platnick and Murphy, 1987) | |
|  | *Eilica modesta* | eilmod | (Keyserling, 1891) | |
|  | *Eilica* sp1 | eilsp1 |  | |
| **Hahniidae** | Hahniidae sp1 | hahsp1 |  | |
|  | Hahniidae sp2 | hahsp2 |  | |
|  | Hahniidae sp3 | hahsp3 |  | |
| **Liniphiidae** | *Agyneta* sp | agynsp |  | |
|  | *Erygone* sp1 | erysp1 | (Miller, 2007) | |
|  | Erygone sp2 | erysp2 | (Miller, 2007) | |
|  | cf *Fissiscapus fractus* | fissic | (Miller, 2007) | |
|  | *Laminacauda montevidensis* | lammon | (Miller, 2007) | |
|  | Linyphiidae indet sp1 | linsp1 |  | |
|  | Linyphiidae indet sp2 | linsp2 |  | |
|  | Linyphiidae indet sp3 | linsp3 |  | |
|  | Linyphiidae indet sp4 | linsp4 |  | |
|  | Linyphiidae indet sp5 | linsp5 |  | |
|  | Linyphiidae indet sp6 | linsp6 |  | |
|  | Linyphiidae indet sp7 | linsp7 |  | |
|  | Linyphiidae indet sp8 | linsp8 |  | |
|  | Linyphiidae indet sp9 | linsp9 |  | |
|  | *Mermessus* sp | mermsp | (Andrei, 2010) | |
|  | *Moyosi prativaga* | moypra | (Miller, 2007) | |
|  | *Neriene* sp | nerien | (Li et al., 2018) | |
|  | *Sphecozone venialis* | sphven | (Miller, 2007) | |
|  | *Tutaibo velox* | tutvel | (Miller, 2007) | |
| **Lycosidae** | *Allocosa* sp | allosp |  | |
|  | Allocosinae sp | allcsp |  | |
|  | *Alopecosa moesta* | alomoe | (Holmberg, 1875) | |
|  | *Geolicosa hyltonscotae* | geohyl | (Mello-Leitão, 1941) | |
|  | *Hogna* sp | hognsp | (O’Neil, 2010) | |
|  | *Lobizon humilis* | lobhum | (Piacentini and Grismado, 2009) | |
|  | *Lobizon minor* | pardsp | (Piacentini and Grismado, 2009) | |
|  | *Lobizon otamendi* | lobota | (Piacentini and Grismado, 2009) | |
|  | *Lycosa erythognatha* | lycery | (Zimber, 1963) | |
|  | *Lycosa* cf *thorelli* | lycfth | (Simó et al., 2002) | |
|  | Lycosa gr thorelli | lyctho | (Simó et al., 2002) | |
|  | Lycosa gr thorelli sp2 | lycsp2 | (Simó et al., 2002) | |
|  | *Lycosa u-album* | lycual | (Mello-Leitão, 1938) | |
|  | *Pardosa flammula* | parfla | (Mello-Leitão, 1945) | |
|  | *Schizocosa malitiosa* | schmal | (O’Neil, 2010) | |
| **Microstigmatidae** | *Xenonemesia platensis* | xenpla | (Ferretti et al., 2010) | |
| **Miturgidae** | *Teminus insularis* | temins | (Platnick and Shadab, 1989) | |
| **Oonopidae** | *Neotrops nigromaculatus* | neonig | (Grismado and Ramírez, 2013) | |
| **Oxyopidae** | *Oxyopes birabeni* | oxybir | (Mello-Leitão, 1941) | |
|  | *Oxyopes salticus* | oxysal | (Chamberlin, 1929) | |
| **Palpimanidae** | *Othiothops birabeni* | othbir | (Mello-Leitão, 1945) | |
| **Philodromidae** | *Tibellus paraguensis* | tibesp | (Achitte-Schmutzler and Rubio, 2016) | |
| **Salticidae** | *Aphirape flexa* | aphfle | (Galiano, 1851) | |
|  | Dendryphantini sp | dendry |  | |
|  | *Habronattus cf paratus* | habpar | (Peckham and Peckham, 1896) | |
|  | *Hisukattus transversalis* | histra | (Galiano, 1987) | |
|  | *Neonella acostae* | neoaco | (Rubio et al., 2015a) | |
|  | *Neonella minuta* | neomin | (Rubio et al., 2015a) | |
|  | *Neonella* cf *nana* | neonan | (Rubio et al., 2015a) | |
|  | *Neonella* sp1 | neosp1 | (Rubio et al., 2015a) | |
|  | *Neonella* sp2 | neosp2 | (Rubio et al., 2015a) | |
|  | Salticidae indet sp1 | salsp1 |  | |
|  | Salticidae indet sp2 | salsp2 |  | |
|  | *Semiopyla* sp | semisp | (Elena and Galiano, 1985) | |
|  | *Sumampattus hudsoni* | sumhud | (Galiano, 1996) | |
|  | cf Trydarssus sp | cftryd |  | |
|  | *Tullgrenella melanica* | tulmel | (Galiano, 1970) | |
|  | *Tullgrenella morenensis* | tulmor | (Galiano, 1970) | |
| **Tetragnathidae** | *Leucauge volupis* | leuvol | (Álvarez-Padilla and Hormiga, 2011) | |
| **Theridiidae** | *Achaearanea* sp | achasp |  | |
|  | *Dipoena sp* | diposp |  | |
|  | *Euryopis spinifera* | eurspi | (Levi, 1963) | |
|  | *Guaraniella mahnerti* | guarsp | (Baert, 1984) | |
|  | *Steatoda ancorata* | steanc | (Levi, 1962) | |
|  | Theridiidae indet sp1 | thesp1 |  | |
|  | Theridiidae indet sp2 | thesp2 |  | |
|  | Theridiidae indet sp3 | thesp3 |  | |
|  | Theridiidae indet sp4 | thesp4 |  | |
|  | Theridiidae indet sp5 | thesp5 |  | |
|  | Theridiidae indet sp6 | thesp6 |  | |
|  | *Theridion* sp | thersp | Levi 1963 | |
|  | *Thymoites piratini* | thypir | Rodrigues & Brescovit 2015 | |
|  | *Thymoites puer* | thypue | Rodrigues & Brescovit 2015 | |
|  | Thymoites sp2 | thysp2 | Levi 1964 | |
|  | Thymoites sp3 | thysp3 | Levi 1964 | |
| **Thomisidae** | *Misumenops maculissparsus* | mismac | Lehtinen & Marusik 2008 | |
|  | Misumenoides sp1 | missp1 |  | |
|  | Misumenoides sp2 | missp2 |  | |
|  | *Synaemops pugilator* | synpug | Mello-Leitão 1941 | |
|  | Thomisidae indet sp1 | thosp1 |  | |
|  | *Tmarus elongatus* | tmaelo | Mello-Leitão 1929 | |
|  | *Tmarus* sp1 | tmasp1 |  | |
|  | *Tmarus* sp2 | tmasp2 |  | |
| **Titanoecidae** | *Goeldia patellaris* | goepat | Simon 1893 | |
| **Trachelidae** | *Meriola cetiformis* | mercet | Platnick & Ewing 1995 | |
| **Zoodariidae** | *Cybaeodamus ornatus* | cyborn | Mello-Leitão 1938 | |

**Reference**

Achitte-Schmutzler, H.C., Rubio, G.D., 2016. First description of the male of the spider Tibellus paraguensis Simon, 1897 (Araneae: Philodromidae), with new distribution records. Zootaxa 4161, 146–150. https://doi.org/10.11646/zootaxa.4161.1.12

Álvarez-Padilla, F., Hormiga, G., 2011. Morphological and phylogenetic atlas of the orb-weaving spider family Tetragnathidae (Araneae: Araneoidea). Zool. J. Linn. Soc. 162, 713–879. https://doi.org/10.1111/j.1096-3642.2011.00692.x

Andrei, V.T., 2010. Order Araneae , family Salticidae. Arthropod fauna UAE 3, 27–69.

Baert, L., 1984. Mysmenidae and Hadrotarsidae from the neotropical Guaraní zoogeographical province (Paraguay and South Brazil) (Araneae). Rev. Suisse Zool. 91, 603–616.

Bonaldo, A.B., 2000. Taxonomy Of The Subfamily Corinninae (Araneae, Corinnidae) In Neotropical And Neartic Regions. Iheringia Ser. Zool. 89, 3–148.

Brescovit, A.D., 1996. Revisão de Anyphaeninae Bertkau a nível de gêneros na Região Neotropical (Araneae, Anyphaenidae). Rev. Bras. Zool. 13, 1–187. https://doi.org/10.1590/s0101-81751996000500001

Chamberlin, R., 1929. On three new spiders of the genus Oxyopes (Araneina). Entomol. news. 40, 17–20.

Edwards, R.J., 1958. The spider subfamily Clubioninae of the United States, Canada and Alaska (Araneae: Clubionidae). Bull. Am. Museum Nat. Hist. 118, 366–434.

Elena, M., Galiano, M.E., 1985. Two new species of Semiopyla with notes on S. cataphracta (Araneae, Salticidae). Rev. suisse Zool. 92, 281–290. https://doi.org/10.5962/bhl.part.81616

Ferretti, N., Pérez-Miles, F., Gonzalez, A., 2010. Mygalomorph Spiders of the Natural and Historical Reserve of Martín García Island, Río de la Plata River, Argentina. Zool. Stud. 49, 481–491.

Galiano, M.E., 1996. Descripción de dos nuevas especies de Salticidae (Araneae) de la Argentina: *Aphirape gamas sp. N.* y *Sumampattus hudsoni sp. N.* Soc. Entomol. Argentina, Buenos Aires (Argentina). 55, 153–159.

Galiano, M.E., 1987. Descripcion de *Hisukattus* nuevo genero (Araneae, Salticidae). Rev. la Soc. Entomol. argentina 44, 137–148.

Galiano, M.E., 1970. Revisión del género Tullgrenella Mello-Leitao (Aranae, Salticidae). Physis 1941.

Galiano, M.E., 1851. Revisión del género *Aphirape* C. L. Koch, 1851 (Aranae, Salticidae). Mus. Argentino ciencias Nat. Bernardino Rivadavia e Inst. Nac. Investig. las ciencias Nat. 1.

Grismado, C.J., Carrión, N.L., 2017. Description of Almafuerte, a new genus of ground spiders from South America (Araneae, Gnaphosidae). Zootaxa 4338, 263–291. https://doi.org/10.11646/zootaxa.4338.2.3

Grismado, C.J., Ramírez, M.J., 2013. The New World goblin spiders of the new genus Neotrops (Araneae: Oonopidae), Part 1. Bull. Am. Museum Nat. Hist. 2013, 1–150.

Holmberg, E.L., 1875. Arácnidos Argentinos. An. Agric. la República Argentina 2, 1998.

Keyserling, G.E., 1891. Die Spinnen Amerikas - Brasilianische Spinnen -. Nürnb. Vv Bauer und Raspe.

Leech, R., 1971. The introduced amaurobiidae of north america, and callobzus hokkaido n. sp. from japan (Arachnida: Araneida). Can. Entomol. 103, 23–32. https://doi.org/10.4039/Ent10323-1

Levi, H.W., 1963. American spiders of the genus *Achaearaneaand* the new genus *Echinotheridion* (Araneae, Theridiidae). Bull. Museum Comp. Zool. 129, 187–240.

Levi, W., 1962. The Spider genera *Steatoda* and *Enoplognatha* in America* (Araneae, Theridiidae). Psyche A J. Entomol. 69, 11–36.

Li, J.Y., Liu, J., Chen, J., 2018. A review of some Neriene spiders (Araneae, Linyphiidae) from China, Zootaxa. https://doi.org/10.11646/zootaxa.4513.1.1

Mello-Leitão, C.F. d., 1945. Arañas de Misiones, Corrientes y Entre Ríos. Rev. del Mus. La Plata 4 Zool., 213–302.

Mello-Leitão, D.C., 1941. Las Arañas de Córdoba, La Rioja, Catamarca, Tucumán, Salta y Jujuy. Colectadas por los profesores Birabén. Rev. del Mus. La Plata 2, 99–198.

Mello-Leitão, D.C., 1938. Algunas arañas nuevas. Rev. del Mus. La Plata 89–118.

Miller, J.A., 2007. Review of Erigonine spider genera in the Neotropics (Araneae: Linyphiidae, Erigoninae). Zool. J. Linn. Soc. 149, 1–263.

O’Neil, M.C., 2010. Las arañas lobo de Uruguay: Taxonomía Y Distribución (Araneae, Lycosidae).

Peckham, G.W., Peckham, E.G., 1896. Spiders of the family Attidae from Central America and Mexico. Nat. Hist. Soc. Wisconsin. https://doi.org/10.1017/CHOL9780521572446.020

Piacentini, L.N., Grismado, C.J., 2009. Lobizon and Navira, two new genera of wolf spiders from Argentina (Araneae: Lycosidae). Zootaxa 33, 1–33. https://doi.org/10.11646/zootaxa.2195.1.1

Platnick, N., Shadab, M., 1989. A Review of the Spider Genus Teminius (Araneae, Miturgidae). Am. Musuem Novit. 12.

Platnick, N., Shadab, M., 1984. A Revision of the Neotropical Spiders of the New Genus Apopyllus (Araneae, Ganphosidae). Am. Musuem Novit. 1–9.

Platnick, N.I., Murphy, J.A., 1987. Studies on Malagasy Spiders, 3. The Zelotine Gnaphosidae (Araneae, Gnaphosoidea), with a Review of the Genus Camillina. Zootaxa 20, 1–33.

Platnick, N.I., Shadab, M.U., 1982. A revision of the American spiders of the genus Drassyllus. Bull. Am. Museum Nat. Hist. 173, 1–97.

Polotow, D., Brescovit, A.D., 2009. Revision and cladistic analysis of isoctenus and description of a new neotropical genus (Araneae, Ctenidae, Cteninae). Zool. J. Linn. Soc. 155, 583–614. https://doi.org/10.1111/j.1096-3642.2008.00452.x

Rubio, G.D., Argañaraz, C.I., Gleiser, R.M., 2015a. A new species of jumping spider neonella gertsch, with notes on the genus and male identification key (Araneae, Salticidae). Zookeys 2015, 1–14. https://doi.org/10.3897/zookeys.532.6078

Rubio, G.D., Zapata, L. V., Grismado, C.J., 2015b. A new species of Castianeira Keyserling (Araneae, Corinnidae) from Buenos Aires, Argentina. Stud. Neotrop. Fauna Environ. 50, 137–143. https://doi.org/10.1080/01650521.2015.1058112

Simó, D., von Eickstedt, D.V.R., 1994. Revisión de la sistemática del género Asthenoctenus Simon, 1897 (Araneae, Ctenidae). Arachnologia 22, 1–12. https://doi.org/10.1017/CBO9781107415324.004

Simó, M., Seguí, R., Pérez-Miles, F., 2002. The Copulatory Organs of the Cryptic Species Lycosa Thorelli and Lycosa Carbonelli and Their Hybrid Progeny, With Notes on Their Taxonomy (Araneae, Lycosidae). J. Arachnol. 30, 140. https://doi.org/10.1636/0161-8202(2002)030[0140:tcootc]2.0.co;2

Zimber, S., 1963. Estudos sóbre aranhas da família Lycosidae(*) 15, 19–24.
