# Supplementary material for: Different land-use types equally impoverish but differentially preserve grassland species and functional traits of spider assemblages
Source: Sci Rep. 2021 May 13;11:10316. doi: 10.1038/s41598-021-89658-7 (PMC8119495; doi:10.1038/s41598-021-89658-7)
Supplement: Supplementary file 2 — Supplementary Information 2. [file 41598_2021_89658_MOESM2_ESM.docx]

**Different land-use types equally impoverish but differentially preserve grassland species and functional traits of spider assemblages**

Carolina M. Pinto^a^*, Pamela E. Pairo^a^, M. Isabel Bellocq^a,b^, Julieta Filloy^a^

^a^Departamento de Ecología, Genética y Evolución, FCEN, Universidad de Buenos Aires – IEGEBA, CONICET, Ciudad Universitaria, Pab 2, piso 4, C1428EHA, Buenos Aires, Argentina.

^b^M. Isabel Bellocq passed away on 9 July 2019.

* Corresponding author. Tel.: +5401156421009.

E-mail address: carolinapinto@ege.fcen.uba.ar - carolinap90@gmail.com (C.M. Pinto)

**Table A2.** Post-hoc contrasts for spider taxonomic and functional dissimilarity three land-use types and seminatural grasslands, *p* < 0.05. N represent the nestedness dissimilarity component and T the turnover dissimilarity component.

| **Pairs** | **Taxonomic dissimilarity** | | **Functional dissimilarity** | |
| --- | --- | --- | --- | --- |
|  | ***F*** | ***p*** | ***F*** | ***p*** |
| Nplantation - Nsoybean | 0.269 | 0.604 | 4.021 | *0.049** |
| Nplantation - Ncattle | 0.237 | 0.633 | 0.002 | 0.965 |
| Nplantation - Tplantation | 1602 | *0.0001** | 1.470 | 0.227 |
| Nplantation - Tsoybean | 1961 | *0.0001** | 0.205 | 0.651 |
| Nplantation - Tcattle | 904.1 | *0.0001** | 1.617 | 0.205 |
| Nsoybean - Ncattle | 1.150 | 0.288 | 4.504 | *0.040** |
| Nsoybean - Tplantation | 1676 | *0.0001** | 0.675 | 0.412 |
| Nsoybean - Tsoybean | 2060 | *0.0001** | 5.856 | *0.019** |
| Nsoybean - Tcattle | 936.8 | *0.0001** | 0.913 | 0.342 |
| Ncattle - Tplantation | 1702 | *0.0001** | 1.611 | 0.211 |
| Ncattle - Tsoybean | 2110 | *0.0001** | 0.284 | 0.593 |
| Ncattle - Tcattle | 932.1 | *0.0001** | 1.831 | 0.183 |
| Tplantation - Tsoybean | 0.782 | 0.377 | 2.716 | 0.107 |
| Tplantation - Tcattle | 1.967 | 0.171 | 0.003 | 0.955 |
| Tsoybean - Tcattle | 4.798 | *0.033** | 3.042 | 0.085 |
