# Supplementary material for: Different land-use types equally impoverish but differentially preserve grassland species and functional traits of spider assemblages
Source: Sci Rep. 2021 May 13;11:10316. doi: 10.1038/s41598-021-89658-7 (PMC8119495; doi:10.1038/s41598-021-89658-7)
Supplement: Supplementary file 3 — Supplementary Information 3. [file 41598_2021_89658_MOESM3_ESM.docx]

**Different land-use types equally impoverish but differentially preserve grassland species and functional traits of spider assemblages**

Carolina M. Pinto^a^*, Pamela E. Pairo^a^, M. Isabel Bellocq^a,b^, Julieta Filloy^a^

^a^Departamento de Ecología, Genética y Evolución, FCEN, Universidad de Buenos Aires – IEGEBA, CONICET, Ciudad Universitaria, Pab 2, piso 4, C1428EHA, Buenos Aires, Argentina.

^b^M. Isabel Bellocq passed away on 9 July 2019.

* Corresponding author. Tel.: +5401156421009.

E-mail address: carolinapinto@ege.fcen.uba.ar - carolinap90@gmail.com (C.M. Pinto)

**Table A3**. NMDS analysis performed to identify the species involved in the taxonomic dissimilarity patterns among land-use types and grasslands habitats. The species in bold showed the strongest association with the land-use types detailed in the table, according to the scores of NMDS1 and NMDS2 axis.

| *Species scores NMDS* | | | |
| --- | --- | --- | --- |
| *Species* | *NMDS1* | *NMDS2* | *Land use* |
| *Larinia* sp | 0.27992163 | 0.22076956 |  |
| ***Metaltella simoni*** | **-0.11200168** | **-0.43201996** | **grassland** |
| Cybaeodamus ornatus | -0.2144786 | 0.05983181 |  |
| *Apopyllus silvestrii* | -0.33496659 | 0.04008656 |  |
| Eilica sp1 | -0.12121409 | -0.5363799 |  |
| *Camillina galianoae* | -0.41432511 | -0.04322655 |  |
| *Camillina pulcher* | 0.14058801 | 0.11693482 |  |
| *Agyneta* sp | -0.09420817 | 0.05571046 |  |
| *Tutaibo velox* | -0.02379528 | -0.03852591 |  |
| Erygone sp1 | 0.27833531 | 0.07410891 |  |
| ***Laminacauda montevidensis*** | **0.12922615** | **0.0418405** | **cattle** |
| Linyphiidae indet sp4 | -0.4348913 | 0.1516804 |  |
| Linyphiidae indet sp7 | -0.4365871 | -0.00887178 |  |
| Linyphiidae indet sp8 | -0.34470901 | 0.02140336 |  |
| Linyphiidae indet sp2 | -0.12811361 | 0.17809445 |  |
| *Sphecozone venialis* | -0.06203233 | -0.02465258 |  |
| Castianeira myrmecotypus | 0.10900339 | 0.23663037 |  |
| *Castianeira coquito* | -0.47685185 | 0.13963087 |  |
| *Falconina gracilis* | -0.08512258 | -0.26884552 |  |
| *Dipoena sp* | 0.38057153 | 0.0908692 |  |
| Thymoites sp3 | 0.32367447 | -0.17565804 |  |
| *Theridion* sp | -0.0029968 | -0.27538776 |  |
| *Thymoites piratini* | 0.22493554 | -0.0738833 |  |
| *Thymoites puer* | -0.15218459 | -0.14237372 |  |
| Thymoites sp2 | -0.40269221 | 0.13507579 |  |
| *Euryopis spinifera* | -0.23612828 | -0.18036524 |  |
| ***Steatoda ancorata*** | **0.48147973** | **0.07829765** | **soybean** |
| *Guaraniella mahnerti* | -0.27434196 | 0.11724248 |  |
| ***Geolicosa hyltonscotae*** | **0.07757055** | **-0.52367894** | **grassland** |
| *Pardosa flammula* | 0.43333617 | 0.18663555 |  |
| ***Lycosa erythognatha*** | **-0.01120173** | **0.20581682** | **cattle** |
| *Lycosa* cf *thorelli* | -0.03598636 | -0.40443119 |  |
| Lycosa gr thorelli | 0.02148788 | -0.2202808 |  |
| Lycosa gr thorelli sp2 | 0.33726479 | -0.03686671 |  |
| *Lobizon humilis* | -0.1249611 | 0.04119314 |  |
| ***Alopecosa moesta*** | **-0.00593733** | **0.27696549** | **cattle** |
| *Sumampattus hudsoni* | -0.13115317 | 0.00574166 |  |
| *Aphirape flexa* | -0.14536457 | -0.22084226 |  |
| Dendryphantini sp | 0.28501593 | -0.12760786 |  |
| *Habronattus cf paratus* | 0.05272282 | -0.51556022 |  |
| ***Hisukattus transversalis*** | **-0.45594858** | **0.21165431** | **plantation** |
| ***Tullgrenella melanica*** | **-0.08654341** | **-0.52724209** | **grassland** |
| ***Tullgrenella morenensis*** | **0.62721192** | **0.22477234** | **soybean** |
| *Neonella acostae* | 0.0826842 | -0.44053345 |  |
| *Neonella* sp1 | 0.19015997 | 0.09610183 |  |
| *Neonella* sp2 | -0.10920827 | -0.25184962 |  |
| *Neonella cf nana* | -0.1533648 | 0.0017721 |  |
| *Semiopyla* sp | -0.13887013 | -0.20803178 |  |
| ***Asthenoctenus borellii*** | **-0.49251026** | **0.34011967** | **plantation** |
| *Oxyopes birabeni* | -0.01737021 | -0.25809383 |  |
| ***Oxyopes salticus*** | **0.23763111** | **0.02497865** | **soybean** |
| *Tibellus paraguensis* | -0.08295334 | -0.22567557 |  |
| Hahniidae sp1 | -0.11465585 | 0.05550626 |  |
| Hahniidae sp2 | -0.22161556 | -0.1032141 |  |
| mercet | 0.18756552 | 0.09480145 |  |
| neonig | -0.07025964 | -0.4876863 |  |
| **tmaelo** | **-0.44124097** | **0.11585472** | **plantation** |
| Thomisidae indet sp1 | 0.23152239 | -0.36638355 |  |
| *Teminus insularis* | -0.07872705 | -0.16646838 |  |
